# Consecutive sessions of transcranial direct current stimulation do not remediate visual hallucinations in Lewy body dementia: a randomised controlled trial

**DOI:** 10.1186/s13195-018-0465-9

**Published:** 2019-01-18

**Authors:** Greg J. Elder, Sean J. Colloby, Michael J. Firbank, Ian G. McKeith, John-Paul Taylor

**Affiliations:** 1 Institute of Neuroscience, Newcastle University, Campus for Ageing and Vitality, Newcastle upon Tyne, NE4 5PL UK; 2 Department of Psychology, Faculty of Health and Life Sciences, Northumbria University, Newcastle upon Tyne, NE1 8ST UK

**Keywords:** Lewy body dementia, Dementia with Lewy bodies, Parkinson’s disease dementia, Transcranial direct current stimulation, Visual hallucinations

## Abstract

**Background:**

Complex visual hallucinations are common in Lewy body dementia (LBD) and can cause significant patient and caregiver distress. Current treatments are primarily pharmacological in nature and have limited efficacy and associated side effects. The objective of this study was to assess the effects of consecutive sessions of transcranial direct current stimulation (tDCS) on visual hallucination frequency and severity in LBD, at short-term and long-term follow-up stages.

**Methods:**

The study was a randomised, double-blind, placebo-controlled trial involving 40 participants with LBD (M_age_ = 75.52 years, SD_age_ = 8.69 years) which was conducted at a single site between November 2013 and December 2017. Participants received two consecutive 20-min sessions of active (0.048 mA/cm^2^) or placebo tDCS, separated by a 30-min break, over 5 consecutive days. The anodal electrode was applied to the right parietal cortex (P4) and the cathodal electrode was applied to the occipital cortex (O_z_). The primary outcome measure was the Neuropsychiatric Inventory (NPI) hallucinations subscale, as completed by a caregiver/informant at baseline and day 5 (short-term) follow-up, and month 1 and month 3 (long-term) follow-up. Secondary outcome measures included visual cortical excitability, as measured using transcranial magnetic stimulation, computerised attentional and visuoperceptual tasks, and measures of global cognition and cognitive fluctuations.

**Results:**

Complete study data were obtained from 36 participants. There was an overall improvement in visual hallucinations (NPI) for both groups at day 5 relative to baseline, with a medium-to-large effect size; however, compared to placebo, active tDCS did not result in any improvements in visual hallucinations (NPI) at day 5 relative to baseline, or at month 1 or month 3 follow-up time points. Additionally, comparisons of secondary outcome measures showed that active tDCS did not result in any improvements on any measure (visual cortical excitability, attentional and visuoperceptual tasks or cognitive measures) at any time point.

**Conclusions:**

Repeated consecutive sessions of parietal anodal tDCS, and occipital cathodal tDCS, do not improve visual hallucinations or visuoperceptual function, or alter visual cortical excitability in LBD.

**Trial registration:**

ISRCTN, ISRCTN40214749. Registered on 25 October 2013.

**Electronic supplementary material:**

The online version of this article (10.1186/s13195-018-0465-9) contains supplementary material, which is available to authorized users.

## Background

Lewy body dementia (LBD) is a term which includes dementia with Lewy bodies (DLB) and Parkinson’s disease with dementia (PDD). DLB is a common cause of degenerative dementia in older people after Alzheimer’s dementia (AD), accounting for up to 7.5% of all diagnosed dementia cases in secondary care [1, 2]; and in Parkinson’s disease (PD), dementia is a common long-term outcome, affecting up to 80% of patients [3]. Individuals with DLB and PDD share a common underlying alpha-synuclein neuropathology and display similar neuropsychological symptoms, including marked attentional and visuoperceptual deficits [4, 5].

Complex visual hallucinations, which are present in up to 80% of LBD patients, are a core symptom for the diagnosis of DLB [2, 6]. The presence of visual hallucinations is associated with behavioural and neuropsychiatric disturbances in individuals; additionally, their presence is associated with an increased likelihood of patient hospitalisation and nursing home admission, and they can negatively impact caregiver distress [7–9]. Currently, treatments for visual hallucinations in DLB and PDD are primarily pharmacological in nature and are typically limited to the use of cholinesterase inhibitors and antipsychotic agents [10]; however, these agents have been shown to have only limited efficacy and, in the case of antipsychotic medications, are typically associated with significant morbidity and mortality risks. There is therefore an urgent need for alternative methods of treatment.

One aetiological theory of visual hallucinations, which is known as the deafferentation hypothesis, suggests that impaired bottom-up processing, from the eye to the primary visual cortex, results in compensatory cortical hyper-excitability and subsequent visual hallucinations [10, 11]. Alternatively, it has been suggested that visual hallucinations occur due to a combination of perceptual and attentional deficits [12–14]. Evidence in support of this latter model comes from work demonstrating an association between visuoperceptual and occipital–parietal deficits in DLB [15] and from neuroimaging perfusion and metabolism studies, which have indicated that underactivity of the posterior parietal cortex is present in DLB and PDD [16, 17]. The parietal cortex has been shown to have a role in attentional processing [18], and it is plausible that parietal hypoactivity contributes to visual hallucinations due to the dorsal visual stream alterations apparent in individuals with DLB who hallucinate [19]. Visual cortical excitability, measured using transcranial magnetic stimulation (TMS), has been shown to positively associate with the frequency and severity of caregiver-rated visual hallucinations in DLB; notably, participants with a greater visuoperceptual deficit displayed a greater level of cortical excitability and more severe visual hallucinations [20]. Additionally, recent neuropathological and magnetic resonance imaging data demonstrate a relative loss of regional visual cortical inhibition in both DLB and PDD respectively [21, 22]. Therefore, the modulation of visual cortical excitability can potentially reduce the frequency and severity of visual hallucinations.

One method by which cortical excitability can be altered is through the use of transcranial direct current stimulation (tDCS), which is an inexpensive, non-invasive brain stimulation technique. This involves the application of a weak electrical current (typically < 2 mA) which is delivered to the brain through scalp electrodes contained within saline or conductive gel-soaked holding bags. The effects of tDCS modulate cortical excitability in a polarity-dependent manner, where anodal stimulation increases and cathodal stimulation reduces the subsequent underlying membrane potential by several millivolts [23, 24]. Studies have demonstrated that tDCS can modulate visual cortical function in a polarity-dependent manner, where anodal stimulation can reduce and cathodal stimulation can increase the TMS-related phosphene threshold [25] and visual-evoked potential [26]; similarly, the application of anodal tDCS to the right posterior parietal cortex has been shown to improve visual search and orienting abilities [27].

To date, no studies have examined the therapeutic use of tDCS as a treatment for visual hallucinations in DLB and PDD, although it has been shown to be both feasible and tolerable in both patient groups [28, 29]. Whilst tDCS can lead to short-term acute effects on cortical excitability, as a single session of approximately 10 min duration can result in after-effects lasting for 1 h [30], repeated stimulation can result in longer-lasting cortical effects persisting beyond 24 h [31]. Notably, the effects of repeated stimulation upon relevant outcomes, including executive function and motor skill acquisition, appear to be additive [32, 33] and it would therefore be expected that the repeated application of tDCS would result in an additive and sustained therapeutic effect.

The aim of the present study was to investigate whether anodal tDCS, applied to the scalp overlying the right parietal cortex, and cathodal tDCS, applied to the midline occiput overlying the visual cortex, reduced the frequency and severity of caregiver-assessed visual hallucinations in DLB and PDD, both at short-term and longer-term follow-up time points. The current study also aimed to examine whether active tDCS reduced visual cortical excitability and improved visuoperceptual function, as secondary outcome measures. Specifically, it was hypothesised that active stimulation, compared to placebo stimulation, would: 1) reduce the frequency and severity of visual hallucinations in the short-term (at day 5) and at long-term (month 1 and month 3) follow-up periods, relative to baseline; 2) would improve visuoperceptual function in the short-term and long-term, relative to baseline; 3) increase the transcranial magnetic stimulation-related phosphene threshold at the short-term follow-up stage, as an objective marker of visual cortical excitability, relative to baseline.

## Methods

### Trial design

The study was a randomised double-blind, placebo-controlled (allocation ratio of 1:1 active/placebo) trial conducted at a single site (Campus for Ageing and Vitality, Newcastle University, Newcastle upon Tyne, UK) between December 2013 and December 2017. Participants completed baseline assessments (day 0), a treatment week (day 1 to day 4), short-term follow-up (day 5) and long-term follow-up (month 1 and month 3) within the trial.

### Participants

Participants who met diagnostic criteria for either probable DLB or PDD [2, 4], as verified by two experienced clinicians, and who were experiencing visual hallucinations of a moderate to severe nature were recruited from clinical services in the North East of England. Participants were included if they: were ≥ 60 years of age; had no changes to relevant anti-parkinsonian or psychotropic medication, or cholinesterase inhibitors, for a period of 1 month prior to participation; had a Mini-Mental State Examination (MMSE) [34] score ≥ 12; and had a sufficient level of English to allow participation.

Exclusion criteria were: relevant skin allergies; a history of excess alcohol intake; concurrent major psychiatric illness; significant physical illness or co-morbidities; other neurological disorders; a history of moderate-to-severe visual impairment secondary to glaucoma, cataract or macular degeneration; or metallic or electronic implants (e.g. pacemakers). The study was prospectively registered (ISRCTN40214749, registered 25 October 2013). All participants and their informants (participant carers/relatives) provided written informed consent and the study was approved by an NHS Research Ethics Committee (Yorkshire & the Humber—Leeds West Research Ethics Committee; REC reference: 13/YH/0292).

### Study settings

**Fig. 1:**
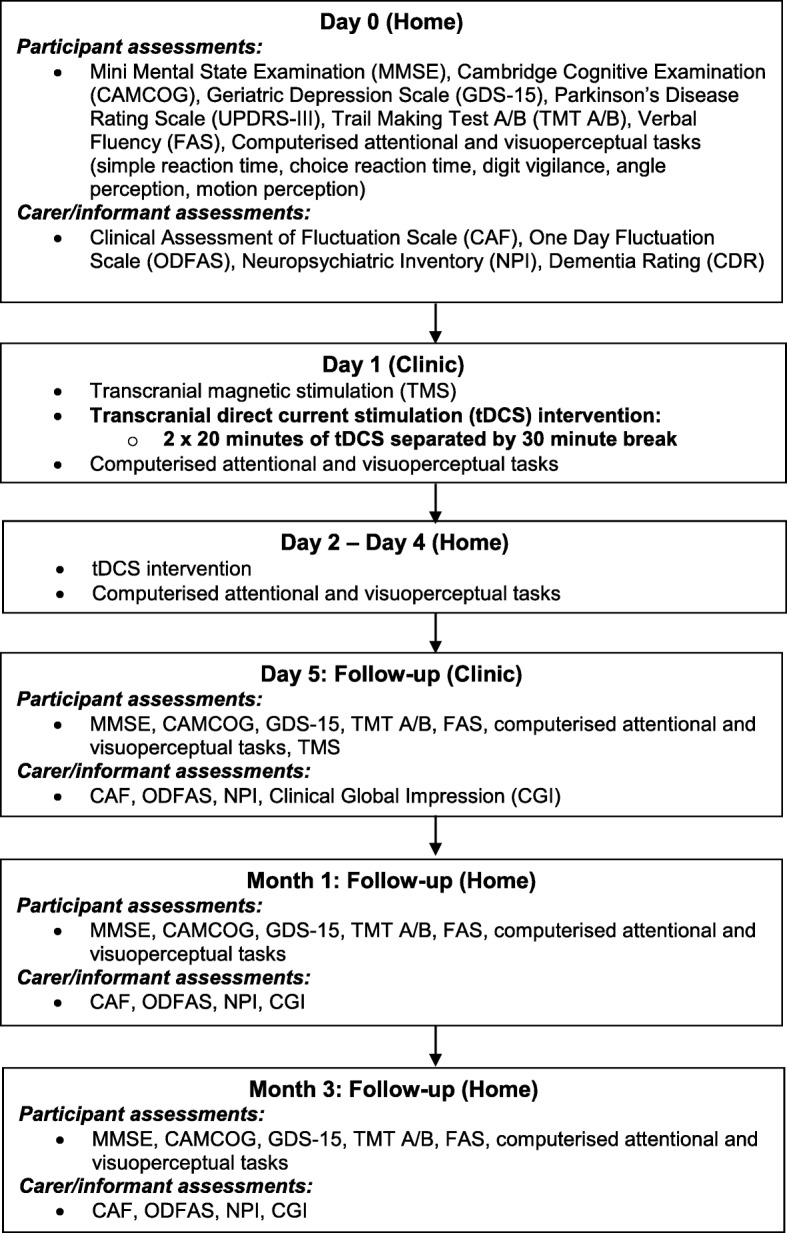
Study procedure

Day 0, day 2–4, month 1 and month 3 procedures were completed in the participant’s usual residence (home or care home environment). Day 1 and day 5 procedures were completed in a clinical research environment. In some situations (e.g. where participants were too frail to travel to the clinical research environment), all study procedures with the exception of TMS were completed in the participant’s usual residence (Fig. 1).

### Measures

#### Cognitive and neuropsychiatric measures

At day 0, cognitive function was measured using the MMSE [34] and the Cambridge Cognitive Examination (CAMCOG) [35]. The presence, and severity, of cognitive fluctuations was assessed using the One Day Fluctuation Scale (ODFAS) and the Clinical Assessment of Fluctuation (CAF) scale respectively [36]. Extrapyramidal motor function was assessed using Part III of the Parkinson’s Disease Rating Scale (UPDRS-III) [37] and the presence of depressive symptoms was assessed using the 15-item Geriatric Depression Scale (GDS-15) [38]. With the help of a carer/informant, the severity of a range of neuropsychiatric symptoms was assessed using the Neuropsychiatric Inventory (NPI) [39]. The Trail Making Test A and B (TMT A/B) [40] and the FAS verbal fluency test [41] were used to assess executive function. Dementia severity was evaluated using the Clinical Dementia Rating (CDR) scale [42] and functional status was assessed using the Instrumental Activities of Daily Living (IADL) scale [43]. Additionally, the Clinical Global Impression (CGI) scale [44] was used to assess the improvement of visual hallucinations relative to baseline at day 5, month 1 and month 3 (CGI—Improvement (CGI-I)).

Visual hallucinations were assessed using the hallucinations sub-scale of the NPI [39], with specific reference to the occurrence of visual hallucinations in order to exclude hallucinations in other modalities (e.g. auditory hallucinations). For reliability purposes, patients were asked whether any visual hallucinations occurred in the previous month using screening questions derived from the North East Visual Hallucinations Inventory III (NEVHI) [45]; discrepancies were discussed with patients and informants, and were used to finalise NPI hallucination scores.

### Computerised attentional and visuoperceptual tasks

**Fig. 2:**
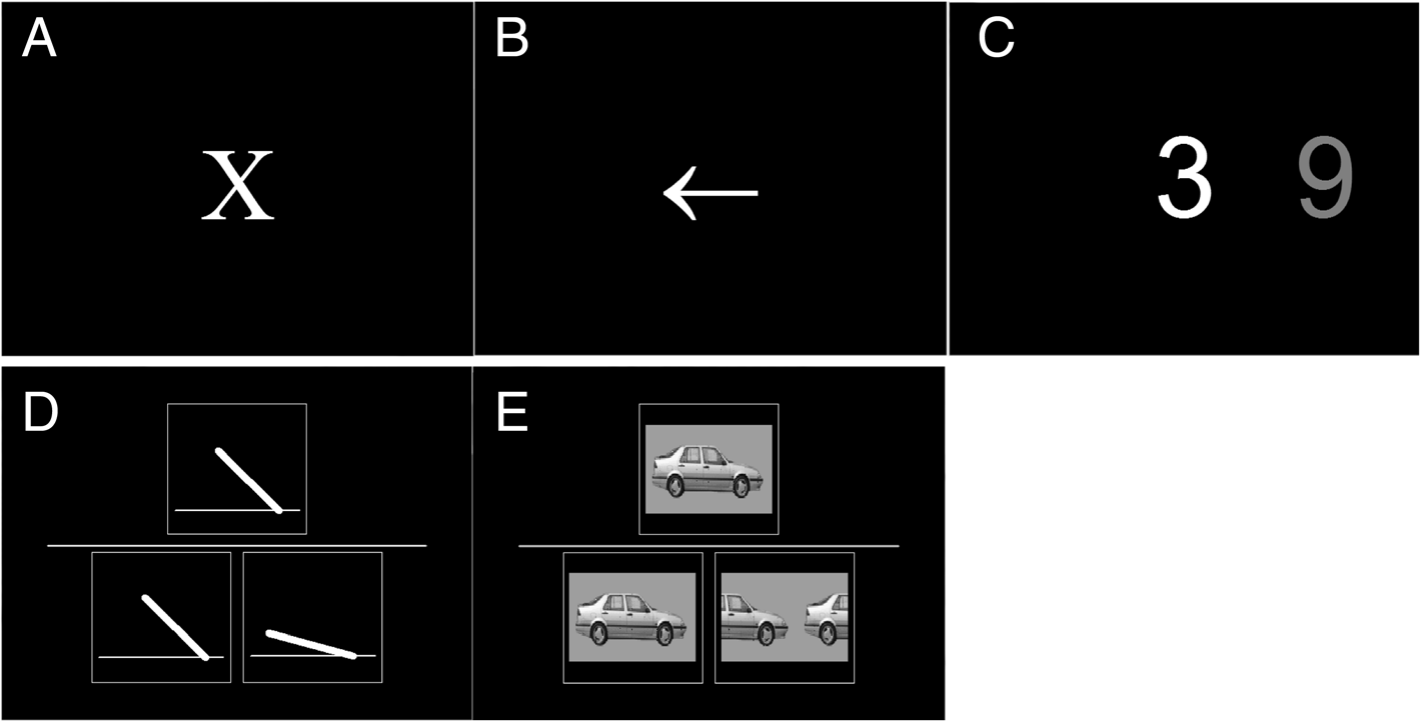
Attentional and visuoperceptual tasks: simple reaction time (**a**); choice reaction time (**b**); digit vigilance (**c**); angle perception (**d**); motion perception (**e**)

Three attentional (simple reaction time (SRT), choice reaction time (CRT), digit vigilance (DV)) and two forced-choice visuoperceptual (angle and motion perception) computerised tasks were used in the current study. These tasks have previously demonstrated that individuals with LBD display a differential performance compared to other dementia groups and healthy individuals [46, 47]. Computerised tasks were presented on a laptop PC and performance was recorded using two custom response buttons, which participants held either in their dominant hand or in both hands depending on the task. Full details of these tasks are provided elsewhere [47–49] and in Fig. 2.

### Occipital transcranial magnetic stimulation

Visual cortical excitability was assessed using a modified and shortened version of a previously reported TMS assessment [20] where participants were required to report the presence or absence of phosphenes immediately following the application of occipital TMS delivered to visual cortical areas.

TMS was delivered using a hand-held single-pulse 70mm figure-of-eight coil connected to a monophasic MagStim 200^2^ stimulator (MagStim, Dyfed, Wales), within a surface latex grid. This consisted of 8 × 8 1cm-spaced points, which was centred on O_z_ and secured to the participant’s scalp. Participants wore an eye mask in order to minimise the potential influence of light adaptation upon the phosphene threshold. Nine grid intersection sites (2cm above, below, left and right of O_z_) were assessed for phosphenes in a pseudo-random order.

The phosphene threshold was determined by increasing the stimulator output from an initial baseline level of 50% to a maximum of 100%, in steps of 10%. Four stimulation trials were given at each output level. If the participant reported a phosphene, the stimulator output was decreased in steps of 1% until the participant no longer reported the presence of phosphenes in the four subsequent consecutive trials. Sham stimulation trials were randomly interspersed with active TMS trials in a 1:8 ratio, where the coil was tilted away from the head at a 90° angle but where one winding remained in contact with the scalp. For sham trials, the stimulator output was set at 100–120% of the phosphene threshold, or at 100% stimulator power if the participant had not reported the presence of a phosphene at that time point. The final phosphene threshold was defined as the threshold (% stimulator output) at which a phosphene was last reported. Non-responders were allocated a phosphene threshold of 101% to allow for the inclusion of their data, in line with previous work [20].

### Intervention

Participants received either active or placebo tDCS. Participants received two consecutive 20-min tDCS sessions, separated by a 30-min break, on 4 consecutive weekdays (Monday–Thursday). Each session of stimulation (1.2 mA) was delivered by HDCStim (Newronika S.R.L., Milan, Italy), using two 25-cm^2^ electrodes soaked in conductive gel (equivalent current density = 0.048 mA/cm^2^). The anodal electrode was placed over the right posterior parietal cortex (P_4_ on the basis of the International 10–20 measurement system [50]) and the cathodal electrode was placed over O_z_. During active stimulation, the current was initially ramped up to a current density of 0.048 mA/cm^2^_,_ in proportional steps, during a 7-s fade-in period. This was followed by a 7-s fade-out period at the end of the stimulation. Impedances were maintained at < 10 kΩ during stimulation. During placebo stimulation, the current was ramped up during an initial 7-s fade-in period, before immediately stopping. Stimulation was administered by a trained technician or research nurse, who were blinded to the stimulation condition. Participants, informants and the assessor were asked whether they thought the participant had received active or placebo stimulation on day 5.

### Sample size calculation

As no studies have assessed the efficacy of tDCS upon visual hallucinations in LBD, sample size calculations were derived from a comparable study [51] where tDCS was used to treat auditory hallucinations in schizophrenia (active *n* = 15, placebo *n* = 15; equivalent current density = 0.057 mA/cm^2^). Participants in the active stimulation group displayed a mean reduction of 31% in the primary outcome measure (Auditory Hallucination Rating Scale), representing a clinical improvement, and the placebo stimulation group displayed an 8% reduction. In the present study, it was determined that the minimum clinically meaningful difference on the primary outcome measure would be a 2-point (16.7%) change in the score between the active and placebo groups at day 5, derived from NPI hallucination frequency × severity scores (maximum score of 12). The pooled standard deviation of 14.05% from Brunelin et al. [51] was used to estimate a minimum effect size of *d* = 1.19.

G*Power 3.1 [52] was used to calculate that 26 participants were required to obtain an effect size of *d* = 1.19 (at 80% power; α = 0.05) and that 52 participants were required to obtain an effect size of *d* = 0.80 (at 80% power; α = 0.05). Therefore, a compromise sample size of 40 was used in order to obtain an expected effect size of *d* = 0.93 (80% power; α = 0.05). Allowing for a 10% participant drop-out rate during the study, this resulted in a final target sample size of 44 participants.

### Randomisation sequence and blinding

Participants were randomised to receive either active or placebo stimulation (1:1 ratio) on the basis of 44 random codes, which were pre-generated by a member of the study team (SJC), in two separate blocks using an online computerised random generator (www.randomization.com). The codes were stored separately from the study site in a sealed opaque envelope. The tDCS stimulator was programmed to deliver either active or placebo stimulation, using an external programming unit (HDCProg; Newronika S.R.L.), by a statistician independent of the study delivery team.

### Statistical analyses

Day 0 (baseline) demographic details were compared between the active and placebo groups using independent-samples *t* tests or Mann–Whitney *U* tests where appropriate. Categorical data were compared using chi-square tests. Effect sizes are provided using Cohen’s *d* or partial eta squared (η^2^_p_).

### Primary outcome measure

The primary outcome measure was the NPI hallucination subscale total score, (frequency × severity), compared between day 0 and day 5, which was analysed using a 2 (treatment group) × 2 (time point) mixed analysis of variance (ANOVA). This analysis was pre-specified. Further exploratory analyses during the follow-up phase compared day 5, month 1 and month 3 NPI-B hallucination scores using a 2 (treatment group) × 3 (time point) mixed ANOVA for participants with complete carer/informant data (*n* = 28). Finally, an exploratory between-group responder analysis was performed using a Fisher exact probability test, where responders were defined as those participants who displayed a ≥ 2-point reduction in NPI-B total (frequency × severity) scores, which was considered to be clinically meaningful, between day 0 and day 5.

### Secondary outcome measures

Analyses of secondary measures were exploratory in nature and only included participants with complete data. TMS phosphene thresholds were compared between day 1 and day 5 using a 2 (treatment group) × 2 (time point) mixed ANOVA. Relevant cognitive and neuropsychiatric measures (CAMCOG, MMSE, CAF, ODFAS) were compared between day 0 and day 5 using a 2 (treatment group) × 2 (time point) mixed ANOVA. Further exploratory analyses during the follow-up phase compared these measures between day 5, month 1 and month 3 using a 2 (treatment group) × 3 (time point) mixed ANOVA. The association between day 0 NPI visual hallucination scores and the day 0 TMS phosphene threshold was examined using a Pearson correlation.

CGI—Improvement scores, measured at day 5, were compared between the active and placebo treatment groups using an independent-samples *t*-test or Mann–Whitney *U* test where appropriate. Further exploratory analyses compared day 5, month 1 and month 3 CGI—Severity and CGI—Improvement scores using a 2 (treatment group) × 3 (time point) mixed ANOVA. The integrity of blinding (participant, carer and assessor) was assessed at day 5 using separate chi-square tests. In order to examine whether there was potentially a differential response to active tDCS, based upon baseline hallucination severity, an additional exploratory analysis investigated whether baseline NPI total scores were different between participants, for both active and placebo conditions, who displayed either an improvement (reduction), no change or a worsening (increase) in NPI scores at day 5 relative to day 0, using a Kruskal–Wallis *H* test.

SRT, CRT and DV task outcome measures included the percentage of correct answers, the mean reaction time (RT) to correct answers and the coefficient of variation (COV), as a marker of intra-individual variability (calculated on the basis of COV = (SD_RT_ / M_RT_) × 100). As reported previously, participant outliers (mean ≥ 2SD) were removed from the SRT, CRT and DV reaction time data [28]. Visuoperceptual angle and motion task outcome measures included the percentage of correct answers and the task difficulty, which was either expressed as degree values (angle task: a lower degree value indicates better task performance) or relative speed values (cars task: a lower relative speed value indicates better task performance). Attentional and visuoperceptual task performance was compared between active and placebo stimulation conditions using a 2 (treatment group) × 6 (measurement time point; day 0–day 5) mixed ANOVA, where the *p*-value was corrected for multiple comparisons. Further exploratory analyses during the follow-up phase compared day 5, month 1 and month 3 attentional and visuoperceptual scores using a 2 (treatment group) × 3 (time point) mixed ANOVA, corrected for multiple comparisons.

Finally, the prevalence of passage and presence hallucinations, assessed on the basis of the NEVHI, were compared qualitatively (i.e. no statistical analysis was conducted) between baseline and day 5.

## Results

### Participant flow

**Fig. 3:**
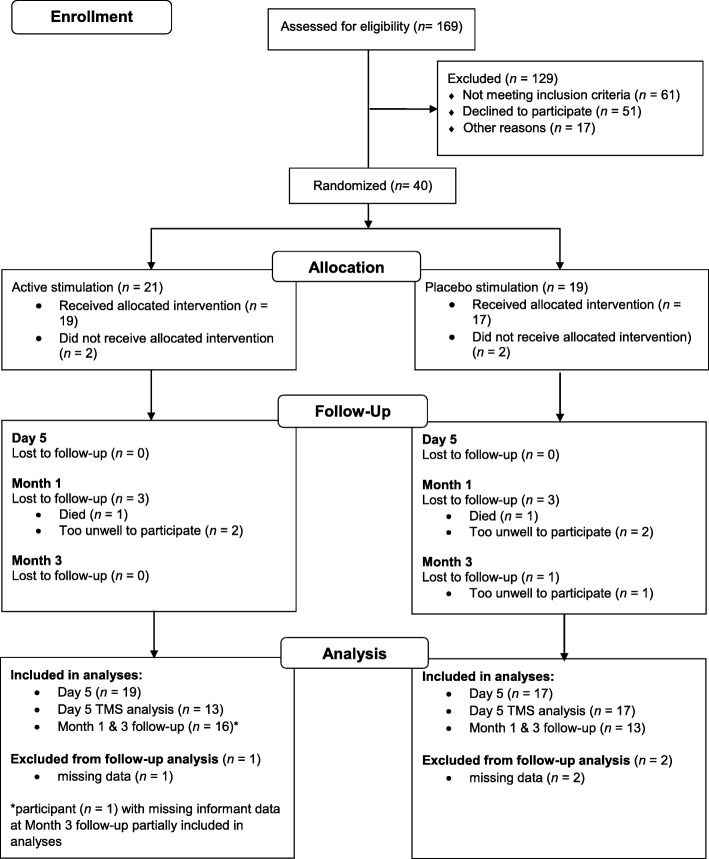
Participant flow diagram

A total of 40 participants (26 DLB, 14 PDD; M_age_ = 75.52 years, SD_age_ = 8.69 years) were entered into the study. Four participants dropped out of the study prior to the treatment week, which resulted in a final treatment week sample of 36 participants (23 DLB, 13 PDD; M_age_ = 75.16 years, SD_age_ = 7.96 years). The mean (± SD) levodopa equivalent dose was 50.00 ± 160.43 mg for DLB participants and 485.50 ± 262.03 mg for PDD participants. Complete TMS data (day 1 and day 5) were obtained for 30 participants; follow-up data were obtained for 30 participants at month 1 and 29 participants at month 3 (Fig. 3). There were no significant between-group differences on any baseline demographic or clinical measure (*p*-values > 0.05; Table 1).

**Table 1:** Baseline participant demographic and assessment data (*n* = 36)

	Active (*n* = 19)		Placebo (*n* = 17)		*p* value
	Mean	SD	Mean	SD	
Age (years)	76.31	8.79	73.88	6.97	0.369
Gender (male/female)	15 (78.9%)/4 (21.1%)		12 (70.6%)/5 (29.4%)		0.563
DLB/PDD	12 (63.2%)/7 (36.8%)		11 (64.7%)/6 (35.3%)		0.923
Levodopa equivalent dose (mg)	225.63	318.79	186.73	264.87	0.695
Cognitive fluctuations, *n* (%)	18 (94.7%)		17 (100%)		0.337
Parkinsonism, *n* (%)	14 (73.7%)		14 (82.4%)		0.532
REM behaviour disorder, *n* (%)	11 (52.4%)		12 (63.2%)		0.491
MMSE	18.16	6.56	17.88	6.06	0.897
CAMCOG (total)	60.05	22.91	58.24	21.39	0.808
NPI hallucinations subscale	4.42	2.80	4.47	3.16	0.961
CAF	6.32	3.92	5.65	2.55	0.553
ODFAS	4.58	3.39	4.71	2.97	0.906
UPDRS-III	36.83	25.07	32.36	21.93	0.561
TMT A (% completion)	52.2%		47.8%		0.923
TMT B (% completion)	5.3%		5.9%		0.935
FAS	17.26	14.75	14.94	11.21	0.602
GDS-15	6.81	4.31	6.27	2.91	0.681
IADL	3.12	2.52	2.47	2.26	0.451
CDR (total)	1.24	0.71	1.29	0.56	0.790

*SD* standard deviation, *DLB* dementia with Lewy bodies, *PDD* Parkinson’s disease dementia, *REM* rapid eye movement, *MMSE* Mini-Mental State Examination, *CAMCOG* Cambridge Cognitive Examination, *NPI* Neuropsychiatric Inventory, *CAF* Cognitive Assessment of Fluctuation, *ODFAS* One Day Fluctuation Scale, *UPDRS-III* Unified Parkinson’s Disease Rating Scale, *TMT* Trail Making Test, *GDS-15* Geriatric Depression Scale (15-item version), *IADL* Individual Activities of Daily Living; *CDR* Clinical Dementia Rating

### Outcomes

#### Primary outcome measure

**Fig. 4:**
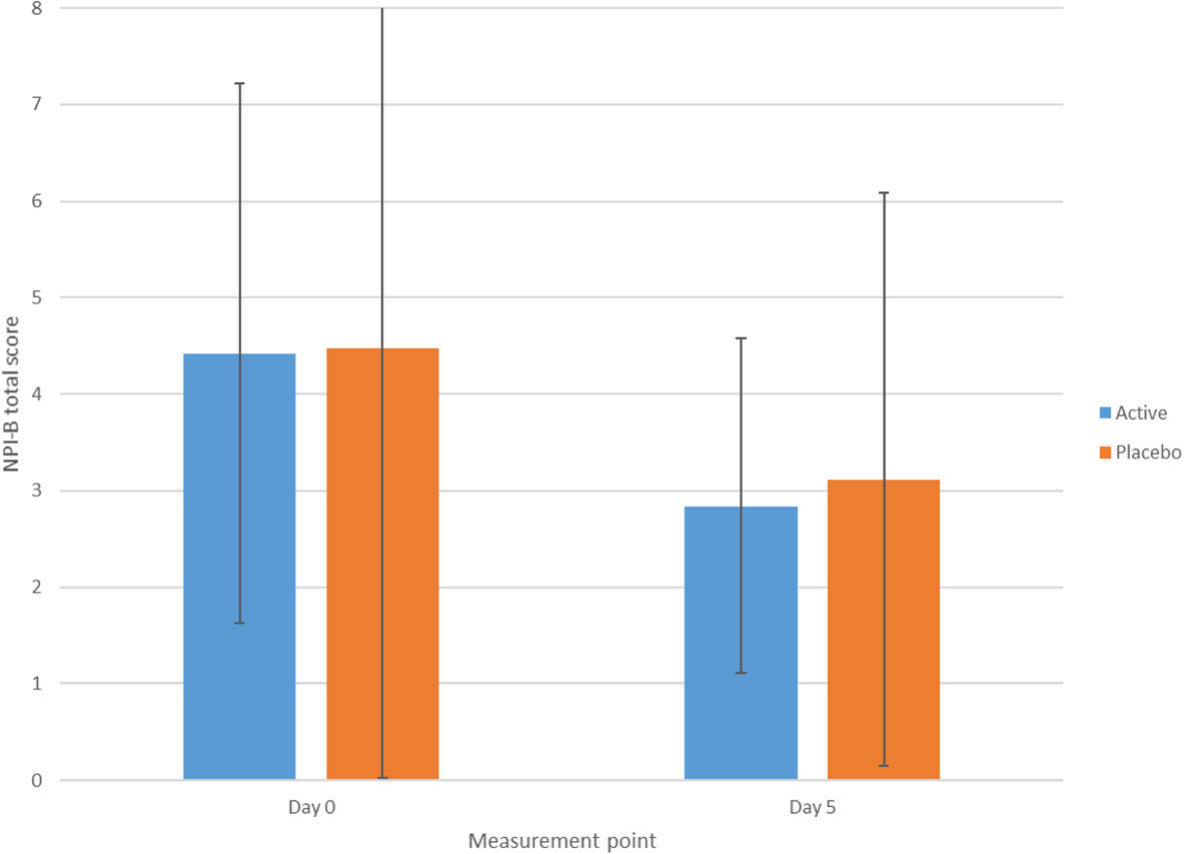
Baseline and day 5 follow-up Neuropsychiatric Inventory (NPI) visual hallucination scores (*n* = 36)

Comparisons of NPI hallucination scores (Fig. 4; Table 2) showed that there were no significant between-group differences between day 0 and day 5, as indicated by the non-significant group × time point interaction, *F*(1,34) = 0.06, *p* = 0.808, η^2^_p_ = 0.002. The main effect of group was not significant, *F*(1,34) = 0.04, *p* = 0.842, η^2^_p_ = 0.00. The main effect of measurement time point was significant, *F*(1,34) = 10.14, *p* = 0.003, η^2^_p_ = 0.23, indicating that both groups showed a significant reduction in visual hallucination severity between day 0 and day 5. Additional analyses indicated that neither the DLB or PDD group alone improved with active stimulation, relative to placebo (Additional file 1).

**Table 2:** Baseline and day 5 comparisons: primary and relevant secondary outcome measures

	Active (*n* = 19)				Placebo (*n* = 17)			
	Day 0		Day 5		Day 0		Day 5	
	Mean	SD	Mean	SD	Mean	SD	Mean	SD
NPI hallucinations subscale	4.42	2.80	2.84	1.74	4.47	3.16	3.12	3.31
CAMCOG	60.05	22.92	59.00	27.23	58.24	21.39	55.12	22.85
MMSE	18.16	6.56	17.42	6.58	17.88	6.06	15.24	6.98
CAF	6.32	3.92	2.95	3.32	5.65	2.55	2.65	2.62
ODFAS	4.58	3.39	3.58	3.04	4.71	2.97	2.71	3.48
TMS phosphene threshold (% output)	73.69	21.83	84.31	23.15	78.88	27.94	89.65	16.33
CGI-I	N/A		3.42	1.26	N/A		3.35	0.93

*SD* standard deviation, *NPI* Neuropsychiatric Inventory, *CAMCOG* Cambridge Cognitive Examination, *MMSE* Mini Mental State Examination, *CAF* Clinical Assessment of Fluctuation, *ODFAS* One Day Fluctuation Scale, *TMS* transcranial magnetic stimulation, *N/A* not applicable, *CGI-I* Clinical Global Impression Scale (Improvement)

Comparisons between day 5, month 1 and month 3 follow-up time points indicated that the group × time point interaction was not significant, *F*(2,52) = 1.05, *p* = 0.357, η^2^_p_ = 0.04. The main effect of group and the main effect of time point were not significant (*p* > 0.05). Additional exploratory analyses indicated that 31 patients (active *n* = 17, placebo *n* = 14) displayed a ≥ 2-point NPI total score improvement, which is considered clinically meaningful, but that this was not significantly different between active and placebo groups, χ^2^(1, *N* = 36), *p* = 0.650.

#### Secondary outcome measures

Relevant cognitive and neuropsychiatric measures (CAMCOG, MMSE, CAF, ODFAS) were compared between day 0 and day 5 (Table 2). Active stimulation did not lead to improvements on any measure at day 5 (all group × time point interaction *p*-values > 0.05). Significant main effects of time point were observed for MMSE, *F*(1,34) = 12.86, *p* = 0.001, η^2^_p_ = 0.27, CAF, *F*(1,34) = 22.00, *p* < 0.001, η^2^_p_ = 0.39, and ODFAS scores, *F*(1,34) = 6.60, *p* = 0.015, η^2^_p_ = 0.16, where decreases were observed for all measures, but not the total CAMCOG score (*p* > 0.05).

The main effect of stimulation condition was not significant for any measure (all *p*-values > 0.05). Active tDCS did not affect the TMS-measured phosphene threshold, as the group × time interaction was not significant, *F*(1,28) = 0.00, *p* = 0.989, η^2^_p_ = 0.00. The main effect of group was not significant, *F*(1,28) = 0.651, *p* = 0.427, η^2^_p_ = 0.02, and the main effect of time point was not significant, *F*(1,28) = 4.16, *p* = 0.051, η^2^_p_ = 0.13. The association between the day 0 TMS phosphene threshold and NPI visual hallucination scores was not significant (*r* = 0.17, *p* = 0.166).

There were no significant differences in CGI-I scores between the active and placebo groups at day 5, *t*(34) = 0.18, *p* = 0.856, *d* = 0.06. Comparisons between day 5, month 1 and month 3 follow-up periods indicated for CGI-I follow-up scores that the group × time point interaction was not significant, *F*(2,52) = 0.09, *p* = 0.915, η^2^_p_ = 0.00, the main effect of group was not significant, *F*(1,26) = 0.11, *p* = 0.749, η^2^_p_ = 0.00, and the main effect of time point was not significant, *F*(2,52) = 1.95, *p* = 0.152, η^2^_p_ = 0.07 (Table 3).

**Table 3:** Day 5, month 1 and month 3 comparisons (*n* = 28)

	Active (*n* = 15)						Placebo (*n* = 13)					
	Day 5		Month 1		Month 3		Day 5		Month 1		Month 3	
	Mean	SD	Mean	SD	Mean	SD	Mean	SD	Mean	SD	Mean	SD
NPI hallucinations subscale	2.80	1.61	3.07	2.52	4.07	2.91	2.92	2.60	3.15	3.29	2.85	2.34
CGI-I	3.40	1.40	3.60	1.45	3.80	1.78	3.15	0.99	3.54	1.33	3.62	1.85

*SD* standard deviation, *NPI* Neuropsychiatric Inventory, *CGI-I* Clinical Global Impression—Improvement

The integrity of blinding at day 5 was maintained for participants, χ^2^(2, *N* = 36) = 0.76, *p* = 0.685, carers/family members, χ^2^(2, *N* = 36) = 0.28, *p* = 0.869, and the assessor, χ^2^(1, *N* = 36) = 3.66, *p* = 0.056.

Day 0 NPI scores were significantly different between participants who showed a day 5 NPI improvement (reduction), no change, or worsening (*H*(2) = 10.19, *p* = 0.006); post-hoc Dunn’s pairwise tests indicated that baseline NPI scores were lower in those patients who worsened, relative to those who improved (*p* = 0.017), indicating that participants with less severe hallucinations worsened and those with more severe hallucinations improved. Day 0 NPI scores were not significantly different for participants in the placebo condition (*p* = 0.799).

Passage hallucinations were present in 16 patients at baseline (44.4%; active *n* = 9, placebo *n* = 7) and 10 patients at Day 5 (27.8%; active *n* = 6, placebo *n* = 4) and presence hallucinations were present in 14 patients at baseline (38.9%; active *n* = 7, placebo *n* = 7) and 5 patients at Day 5 (13.9%; active *n* = 2, placebo *n* = 3); qualitatively, this indicated that the presence or absence of these phenomena did not change following stimulation.

There were no significant between-group changes in attentional (SRT, CRT, DV) or visuoperceptual (angle, motion) function between day 0 and day 5, as there were no significant group × time interactions, main effects of time point or group for any measure (all corrected *p*-values > 0.0038; Table 4). Similarly, there were no significant between-group changes when day 5, month 1 and month 3 follow-up data were examined (all main effects and interaction corrected *p*-values > 0.0038).

**Table 4:** Attentional and visuoperceptual task comparisons (day 0 vs day 5)

	Active *n* = 19				Placebo *n* = 17			
	Day 0		Day 5		Day 0		Day 5	
	Mean	SD	Mean	SD	Mean	SD	Mean	SD
Attentional tasks								
SRT: correct answers (%)^a^	94.51	6.87	91.76	14.34	89.05	15.49	85.48	22.29
SRT: mean RT (ms), correct answers^b^	542.36	213.12	501.36	182.91	760.89	535.01	593.98	185.55
SRT: coefficient of variation (%)^a^	51.99	30.36	52.17	31.54	80.83	41.47	74.17	51.63
CRT: correct answers (%)^c^	79.26	23.11	81.85	26.82	69.11	34.19	79.33	22.75
CRT: mean RT (ms), correct answers^d^	863.29	333.03	808.24	218.21	946.87	260.95	1059.19	576.05
CRT: coefficient of variation (%)^e^	56.86	35.59	44.83	29.18	46.68	27.93	56.56	40.12
DV: correct answers (%)^f^	65.45	24.11	63.37	24.73	59.07	24.92	60.37	21.76
DV: mean RT (ms), correct answers^g^	676.68	97.96	675.88	135.74	683.95	96.12	661.17	125.49
DV: coefficient of variation (%)^h^	29.90	12.23	29.77	14.08	32.21	14.56	41.27	18.68
Visuoperceptual tasks								
Angle: correct answers (%)^i^	72.92	21.84	74.17	15.23	70.83	19.23	70.00	14.56
Angle: difficulty (degrees)^i^	41.34	31.79	39.89	30.47	48.48	34.65	47.87	30.83
Motion: correct answers (%)^j^	53.81	17.97	55.95	16.29	55.28	15.86	56.11	10.72
Motion: difficulty (relative speed)^j^	3.57	0.89	3.49	0.97	3.66	0.71	3.91	0.12

*SD* standard deviation, *SRT* simple reaction time, *RT* reaction time, *CRT* choice reaction time, *DV* digit vigilance

^a^Active *n* = 17, placebo *n* = 14 (due to incomplete data or removal of RT > 2SD)

^b^Active *n* = 15, placebo *n* = 12 (due to incomplete data or removal of RT > 2SD)

^c^Active *n* = 18, placebo *n* = 15 (due to incomplete data or removal of RT > 2SD)

^d^Active *n* = 16, placebo *n* = 11 (due to incomplete data or removal of RT > 2SD)

^e^Active *n* = 18, placebo *n* = 13 (due to incomplete data or removal of RT > 2SD)

^f^Active *n* = 16, placebo *n* = 15 (due to incomplete data or removal of RT > 2SD)

^g^Active *n* = 15, placebo *n* = 10 (due to incomplete data or removal of RT > 2SD)

^h^Active *n* = 16, placebo *n* = 13 (due to incomplete data or removal of RT > 2SD)

^i^Active *n* = 16, placebo *n* = 12 (due to incomplete data or removal of RT > 2SD)

^j^Active *n* = 14, placebo *n* = 12 (due to incomplete data or removal of RT > 2SD)

### Safety and tolerability

All participants tolerated stimulation and, other than a brief tingling sensation underneath the electrodes, no adverse events were reported during stimulation. One patient was hospitalised on day 4 (due to a fall) and two patients died prior to the month 1 follow-up assessment. However, all cases were judged to be unrelated to tDCS (the causes of death were withdrawal of life-maintaining care and pneumonia respectively).

## Discussion

This randomised, placebo-controlled, double-blind trial demonstrated that two 20-min sessions of anodal tDCS, delivered to the right parietal cortex, and cathodal tDCS, delivered to the occipital cortex, repeated over 4 consecutive days, did not reduce the frequency and severity of visual hallucinations in LBD patients. This was observed to be the case at short-term (day 5) and longer-term (month 1 and month 3) follow-up time points, relative to baseline. Additionally, the administration of tDCS did not reduce visual cortical excitability (measured at day 5) or visuoperceptual or attentional function, either at short-term or long-term follow-up time points. Overall, these results suggest that tDCS had no therapeutic effect on visual hallucinations, or on cognitive functions which are implicated in the aetiology of visual hallucinations [12–14], at either short-term or long-term follow-up periods. However, to our knowledge, this is the first study to demonstrate that repeated sessions of tDCS delivered consecutively over multiple days is both feasible and tolerable in an LBD population.

There are a number of reasons which might explain the negative findings observed in the present study. Although the current electrode configuration was chosen on the basis of aetiological models which posit that visual hallucinations arise from a combination of perceptual and attentional deficits [12–14], and from work demonstrating that parietal hypoactivity is a feature of DLB [15–17] and that the parietal cortex is involved in attentional processing [18], this tDCS configuration may not be optimal. Frontal areas are also believed to have a role in the aetiology of visual hallucinations [10, 53]; therefore, the dorsolateral prefrontal cortex (DLPFC) may be an alternative therapeutic target, given the role of the DLPFC in top-down cognition [54] and from previous work demonstrating that the application of tDCS to the DLPFC can benefit attention [55]. Alternatively, a combination of tDCS and behavioural paradigms may reinforce or improve bottom-up processing; for example, the combination of tDCS and focusing attention on visual hallucinations.

Despite our use of repeated stimulation sessions, delivered consecutively over multiple days, which has been shown to result in additive effects [32, 33], other stimulation parameters, including the current density and electrode type, may need to be adjusted in order to maximise the efficacy of tDCS for visual hallucinations. Whilst the current density used in the present study (0.048 mA/cm^2^) is broadly in alignment with other relevant positive studies [27, 51], higher current densities might be required in order to induce a positive effect. Whilst previous LBD studies have indicated that a single session of tDCS at higher densities (0.08 mA/cm^2^) is well tolerated [28, 29], it is not known whether repeated densities, at multiple time points over consecutive days, would be equally well tolerated. The current flow of tDCS can also be affected by structural brain changes [56], and atrophy, particularly parietal and visual cortical atrophy, is a feature of LBD [57]. However, a limitation of the present study was that participants did not routinely undergo magnetic resonance imaging prior to participation and therefore the extent of any atrophy was not known. The modality of stimulation may also be a factor; repetitive TMS has an established evidence base in the treatment of a range of neuropsychiatric symptoms [58] and, compared to tDCS, has the advantage of improved focality [59]. That said, potential disadvantages of TMS in a clinical context include the time-consuming and technical nature of delivery [60]. However, recent developments in the delivery of tDCS, such as high-density electrodes, which might further enhance cortical excitability and result in longer-lasting effects [61], or the application of computational techniques to accurately model current flow [62], may improve the therapeutic utility of tDCS. Given the relative ease of application, low-cost nature and potential clinical applications of tDCS within a home environment, further work should first examine whether an increased current density or use of high-density electrodes can result in a therapeutic effect in LBD.

Although active tDCS did not improve visual hallucinations in LBD, both groups displayed a significant overall reduction in informant-rated visual hallucinations at day 5 relative to baseline, accompanied by a medium-to-large effect size, irrespective of treatment. Similarly, informant-rated short-term (ODFAS) and longer-term (CAF) cognitive fluctuations improved. Global cognition, measured using the MMSE, worsened between the same time points. Therefore, participation in a demanding clinical trial with multiple assessments might benefit some informant-based clinical outcomes, but worsen objective performance, potentially due to fatigue. This has implications for future LBD trials because specific elements of research participation might benefit patients; this may include social contact, changes in routine or involvement in cognitively stimulating activities. Future trials should also identify participants who might respond to interventions prior to trial participation; this approach has been successfully used in a study examining the efficacy of pimavanserin, a selective 5HT2A antagonist, in PD psychosis, where non-specific psychosocial therapies were used to screen participants with neuropsychiatric symptoms [63]. In the present study, the majority of participants displayed a clinically meaningful improvement in NPI scores; however, among these patients, those with more severe hallucinations improved and those with less severe hallucinations worsened, which may reflect regression to the mean. Therefore, large inter-individual variations in neuropsychiatric symptoms in LBD appear to be normal and an awareness of this is important for future interventional trial design in this patient group.

The main strength of the current study is in the double-blind, placebo-controlled nature of the study, as tDCS studies with dementia populations typically lack a placebo group [64]. One potential limitation is in the use and validity of informant-driven visual hallucination assessment scales; whilst the NPI is widely used for assessing symptom severity in clinical trials, sensitive patient-led measurement tools are needed to overcome potential issues with regards to accuracy and reliability. This includes the reluctance of some patients to disclose or fully describe their visual hallucinations, and the fluctuations in insight exhibited by patients with greater impairment [10], which can markedly affect informant accuracy in judging visual hallucination frequency and severity. Objective biomarkers of VH severity are needed, and the pareidolia test might be one such valid and reliable surrogate measure of visual hallucinations [65]. Whilst a potential limitation of the present study is in the concurrent use of medications such as cholinesterase inhibitors, memantine and levodopa [66–68], no between-group differences were observed and medication withdrawal prior to study entry was likely to have resulted in the clinical deterioration of participants.

In conclusion, consecutive daily sessions of anodal tDCS to the parietal cortex, and cathodal tDCS to the occipital cortex, do not benefit visual hallucinations or visuoperceptual performance, or alter visual cortical excitability, in LBD. However, intensive and demanding clinical trials are feasible in an LBD population, and participation may result in relevant short-term clinical benefits.

## Additional file

Additional file 1: Supplementary analyses. (DOCX 14 kb)
