# Supplementary material for: Consecutive sessions of transcranial direct current stimulation do not remediate visual hallucinations in Lewy body dementia: a randomised controlled trial
Source: Alzheimers Res Ther. 2019 Jan 18;11:9. doi: 10.1186/s13195-018-0465-9 (PMC6339360; doi:10.1186/s13195-018-0465-9)
Supplement: Supplementary file 1 — Supplementary analyses. (DOCX 14 kb) [file 13195_2018_465_MOESM1_ESM.docx]

Additional file 1: Supplementary analyses

In order to examine whether the DLB or PDD group showed a differential response to active or placebo treatment, the NPI hallucinations subscale was also compared between baseline and Day 5, using a three-way (2 × 2 × 2) mixed ANOVA. The diagnosis × group × time point interaction was not significant, *F*(1, 32) = .51, *p* = .480, η^2^_p_ *=* .02, indicating that neither group showed a differential response. Neither the time point × stimulation condition interaction, nor the time point × diagnosis interaction, was significant (*p­*-values > .05), and the main effect of time point was significant, which indicated an overall improvement in hallucinations between baseline and Day 5, as discussed in the main text.

The effects of active stimulation upon delusions, as measured using Section A of the NPI, were also explored using a 2 × 2 mixed ANOVA. This indicated that there was a main effect of time point, indicating an overall improvement in delusions between baseline and Day 5, as discussed in the main text. However, the time point × stimulation interaction was not significant, *F*(1,34) = .18, *p* = .175, η^2^_p_ *=* .05, demonstrating that stimulation did not affect delusions.
